# Quantitative MRI of skeletal muscle in a cross‐sectional cohort of patients with spinal muscular atrophy types 2 and 3

**DOI:** 10.1002/nbm.4357

**Published:** 2020-07-18

**Authors:** Louise A.M. Otto, W‐Ludo van der Pol, Lara Schlaffke, Camiel A. Wijngaarde, Marloes Stam, Renske I. Wadman, Inge Cuppen, Ruben P.A. van Eijk, Fay‐Lynn Asselman, Bart Bartels, Danny van der Woude, Jeroen Hendrikse, Martijn Froeling

**Affiliations:** ^1^ Department of Neurology, UMC Utrecht Brain Center, University Medical Center Utrecht Utrecht University the Netherlands; ^2^ Department of Neurology BG‐University Hospital Bergmannsheil, Ruhr‐University Bochum Bochum Germany; ^3^ Department of Neurology and Child Neurology, UMC Utrecht Brain Center University Medical Center Utrecht, Utrecht University the Netherlands; ^4^ Biostatistics & Research Support, Julius Center for Health Sciences and Primary Care University Medical Center Utrecht, Utrecht University Utrecht The Netherlands; ^5^ Department of Child Development and Exercise Center University Medical Center Utrecht, Utrecht University the Netherlands; ^6^ Department of Radiology University Medical Center Utrecht, Utrecht University the Netherlands

**Keywords:** diffusion tensor imaging, magnetic resonance imaging, quantitative imaging, skeletal muscle, spinal muscular atrophy

## Abstract

The aim of this study was to document upper leg involvement in spinal muscular atrophy (SMA) with quantitative MRI (qMRI) in a cross‐sectional cohort of patients of varying type, disease severity and age. Thirty‐one patients with SMA types 2 and 3 (aged 29.6 [7.6‐73.9] years) and 20 healthy controls (aged 37.9 [17.7‐71.6] years) were evaluated in a 3 T MRI with a protocol consisting of DIXON, T2 mapping and diffusion tensor imaging (DTI). qMRI measures were compared with clinical scores of motor function (Hammersmith Functional Motor Scale Expanded [HFMSE]) and muscle strength. Patients exhibited an increased fat fraction and fractional anisotropy (FA), and decreased mean diffusivity (MD) and T2 compared with controls (all *P* < .001). DTI parameters FA and MD manifest stronger effects than can be accounted for the effect of fatty replacement. Fat fraction, FA and MD show moderate correlation with muscle strength and motor function: FA is negatively associated with HFMSE and Medical Research Council sum score (τ = −0.56 and −0.59; both *P* < .001) whereas for fat fraction values are τ = −0.50 and −0.58, respectively (both *P* < .001). This study shows that DTI parameters correlate with muscle strength and motor function. DTI findings indirectly indicate cell atrophy and act as a measure independently of fat fraction. Combined these data suggest the potential of muscle DTI in monitoring disease progression and to study SMA pathogenesis in muscle.

## Graphical abstract

qMRI of thigh muscle in a cross‐sectional cohort of spinal muscular atrophy patients reveals an increased fat fraction and fractional anisotropy (FA), and decreased mean diffusivity (MD) and T2 compared with controls. We acknowledged the confounding effect of fatty infiltration on our data with simulations. DTI parameters FA and MD manifest stronger effects than can be accounted for by the effect of fat replacement. DTI findings indirectly indicate cell atrophy and act as a measure independently of fat fraction.

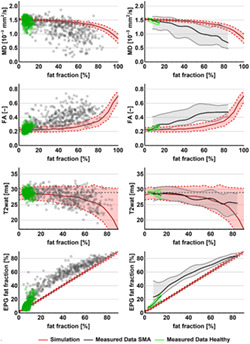

**Abbreviations used**
DMD: Duchenne muscular dystrophy
DTI: diffusion tensor imaging
FA: fractional anisotropy
HFMSE: Hammersmith Functional Motor Scale Expanded
LGMD: limb girdle muscular dystrophies
MD: mean diffusivity
qMRI: quantitative MRI
SMA: spinal muscular atrophy

## 1 INTRODUCTION

Hereditary proximal spinal muscular atrophy (SMA) is caused by survival motor neuron (SMN) protein deficiency due to homozygous loss of function of the *SMN1* gene.^1^ The presence of the nearly identical *SMN2* gene in the human genome ensures production of residual levels of full length SMN protein and the *SMN2* copy number inversely correlates with disease severity.^2^ Treatment strategies that aim to increase SMN protein levels have been shown to be effective, in particular in infants and younger children with SMA.^3^ Treatment of patients with the antisense oligonucleotide Spinraza, which skews splicing of *SMN2* towards full length transcripts, improves survival and motor milestone achievement in children with infantile onset SMA (ie, type 1) and motor function in SMA type 2.^4^ Additional data are needed to judge the efficacy of SMN‐augmenting strategies in older children and adults.

Levels of SMN protein are most critical in alpha‐motor neurons, but other tissues, including muscle, probably also require specific threshold levels for proper development and function.^5^, ^6^ Clinical studies have documented that the natural history of SMA is characterized by decline in muscle strength and motor function over time after an initial phase of stalled gross motor development in early childhood.^7^ Less is known of the natural history of anatomy and function of the tissues constituting the motor unit, ie, the motor neuron, neuromuscular junction and muscle. Degeneration of alpha‐motor neurons, which is the classical pathological hallmark of SMA, can be monitored in vivo by nerve conduction study techniques, in particular compound muscle action potential amplitude.^8^ Few studies have addressed anatomy and function of muscle over time. Magnetic resonance imaging (MRI) is a powerful and noninvasive tool to study anatomy and tissue characteristics of skeletal muscle in vivo.^9^ Most imaging studies in SMA have focused on a qualitative appreciation of muscle, grading fat infiltration visually.^10^, ^11^, ^12^, ^13^, ^14^, ^15^, ^16^, ^17^, ^18^, ^19^, ^20^ Quantitative MRI (qMRI) can complement qualitative evaluation of muscles and has shown promising results in other neuromuscular disorders, such as Duchenne muscular dystrophy (DMD) and limb girdle muscular dystrophies (LGMD).^21^, ^22^ Quantitative MRI in SMA has not been studied in detail.^16^, ^23^, ^24^ The application of qMRI in diseased muscle proposes some technical challenges, as T2 signal and DTI parameters can be influenced or are confounded by fat present in the muscle.^21^, ^25^, ^26^

This study aims to document qMRI properties of muscle tissue in SMA. To this end, we explore the feasibility of a scan protocol of the upper leg that includes DIXON, T2 mapping and diffusion tensor imaging (DTI) in a cross‐sectional cohort of 31 patients with SMA types 2 and 3, encompassing a broad range of disease severity and disease duration. We present the distinctive patterns of fatty infiltration and the associations of qMRI measurements with clinical parameters. We also assessed bias of fat infiltration for the interpretation of magnetic resonance (MR) metrics in the presence of severe fat infiltration that is a hallmark of neuromuscular pathology.

## 2 METHODS

### 2.1 Study population

We recruited patients through the Dutch SMA database. Patients who agreed to participate were asked to recruit an age‐ and gender‐matched control. The diagnosis SMA was genetically confirmed in all patients. We used the clinical classification system for SMA typing^2^: the highest acquired milestone of “independent sitting” for type 2, “walk independently at any stage in life” for type 3 and symptom onset before (3a) or after 36 months (3b), yet before adulthood. In any case of discrepancy between the age at onset or the highest acquired motor milestone, the latter defined the SMA type.

Exclusion criteria were: tracheostomy, tracheostomal or any type of invasive ventilation; forced vital capacity (FVC) > 15% postural change between sitting and supine or symptoms of nocturnal hypoventilation; presence of pronounced swallowing disorders; orthopnea; contra‐indication for 3 T MR or non‐MR compatible material in the body; pregnancy; claustrophobia.

The study protocol was approved by the local ethics committee (no. 17–226/NL61066.041.17). All participants, and/or their parents in the case of minors, had to be capable of understanding the study information and had to give oral and written informed consent.

### 2.2 Clinical evaluation

All subjects underwent a clinical evaluation of muscle strength of 42 muscle groups using the Medical Research Council (MRC) score, which is a widely used scoring system to document the muscle strength of separate muscles.^27^ Motor function was assessed with the Hammersmith Functional Motor Scale Expanded (HFMSE) (range 0‐66). A lower score on the HFMSE indicates poorer motor ability and function. Testing was performed by two trained evaluators (LO and DvdW) on the same day, either prior to MR with 1 hour of nonstraining exercise before scanning, or following MR examination. Lung function in patients was measured by FVC in the sitting and lying position prior to the MR examination.

### 2.3 MR acquisition

All MR examinations were performed on a 3 T MR scanner (Philips Ingenia, Philips Medical Systems, the Netherlands). Subjects were scanned in the supine position, feet‐first with a 12‐channel posterior and 16‐channel anterior body coil, and field of view (FOV) set at 17.5 cm distance from the upper limit of the femoral head stretching 15 cm towards the knee. In children and subjects of smaller statue and/or hindered by severe contractures, the image stack was centered mid‐femoral. Both legs were scanned.

**FIGURE 1:**
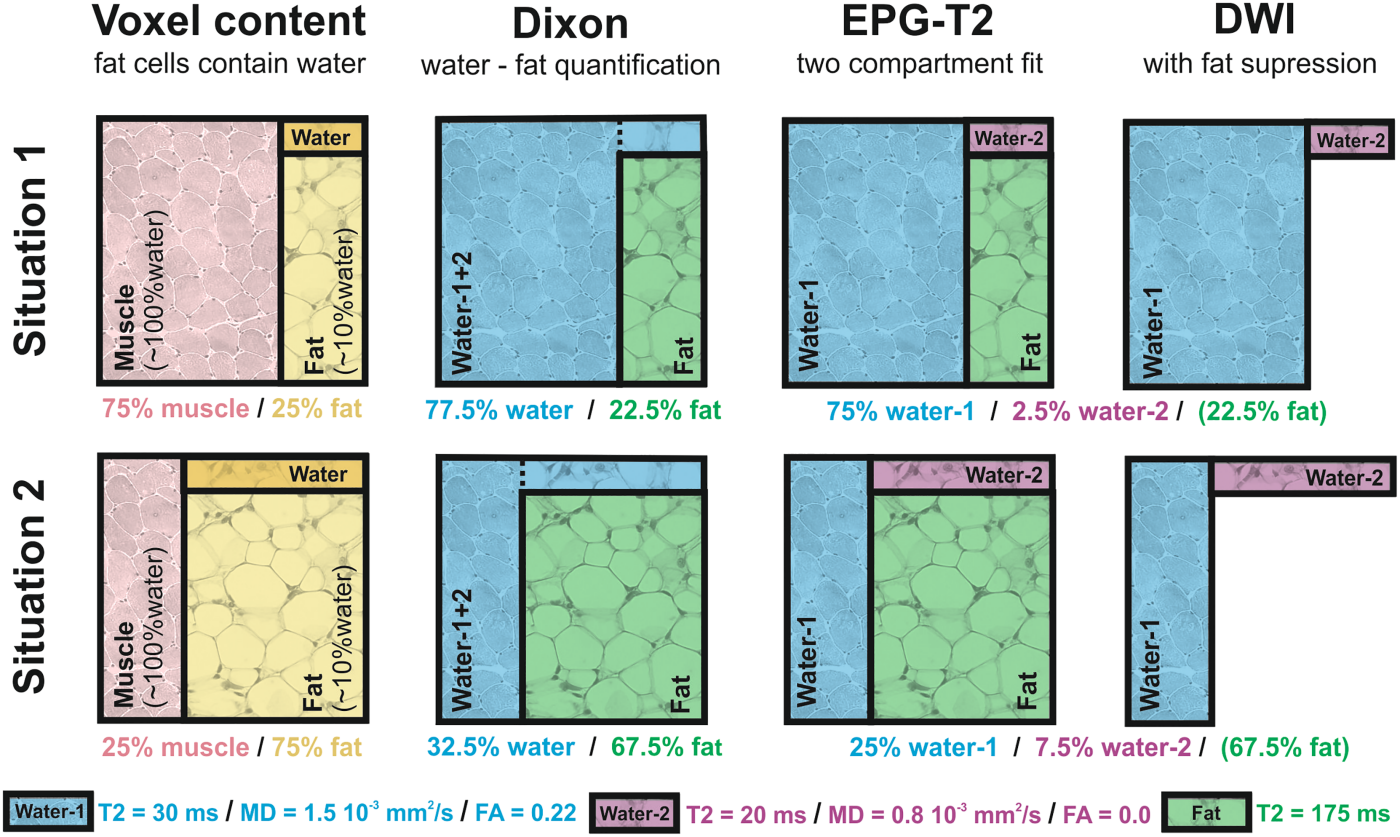
Methods of DIXON, EPG‐T2 and DWI explained. A schematic representation of the water‐fat partial volume effect in each of the used acquisition methods. Situation 1 (top row) represents a voxel with low fat infiltration and situation 2 (bottom row) one with high fat infiltration. The pink quadrant in the first column represents muscle tissue, which is assumed to contain only water. The yellow quadrant represents fat tissue, which is assumed to contain ~10% of water that is rendered dark yellow. The DIXON method distinguishes between water and fat, and slightly underestimates the fat quantity because of attributing its water content to the water/muscle compartment. EPG‐T2 uses a two‐compartment fit accounting for the fat compartment. However, the estimated T2 of the water compartment is a combination of water in muscle and water in fat. The diffusion‐weighted imaging (DWI) acquisition has fat suppression and therefore only measures the water signal. Therefore, the measured diffusion properties are a combination of water in muscle and water in fat

Total scanning time was ~10 minutes including the survey and comprised three examinations, ie, a DIXON acquisition for measuring fat infiltration, T2 mapping to determine the water T2 relaxation time and DTI scans to measure the hindered diffusion of water molecules in tissue. For each voxel, the rate of diffusion and a preferred direction oriented in a three‐dimensional space can be obtained. Fractional anisotropy (FA) describes the anisotropic organization of tissue, approaching 1 when the diffusion is mainly aimed in one direction. Mean diffusivity (MD) is the average diffusion in all directions. MD combined with FA gives insight into the microstructure of tissue.^28^ Acquisition parameters of the MR protocol are listed in Table 1. The acquisition methods are further explained in Figure 1.

**TABLE 1:** Acquisition parameters of MR protocol. Specifications per sequence at a field strength of 3 T

Sequence	4‐point DIXON	T2 mapping	DWI
Sequence	Multi acquisition gradient echo	multi echo spin echo	spin echo‐EPI
Repetition time (ms)	210	4598	5000
Echo time (ms)	2.6/3.36/4.12/4.88	17 x 7.6	57
Flip angle (degrees)	10	90/180	90/180
Acquisition matrix	320 x 320		160 x 92
FOV	480 x 480		480 x 276
Resolution (mm^2^)	1.5 x 1.5	3 x 3	3 x 3
Slices	25	13	25
Slice thickness (mm)	6	6	6
Slice gap (mm)	0	6	0
b‐values (number of images) (mm/s^2^)		0	0 (1), 1 (6), 10 (3), 25 (3), 100 (3), 200 (6), 400 (8), 600 (12)
Fat suppression			Gradient inversion + SPAIR (main fat signal) + SPIR (olefinic fat signal)
SENSE/Partial Fourier	2/1	2/1	1.9/0.75
Acquisition time (min:s)	1:20	3:05	3:30

### 2.4 MR processing

**FIGURE 2:**
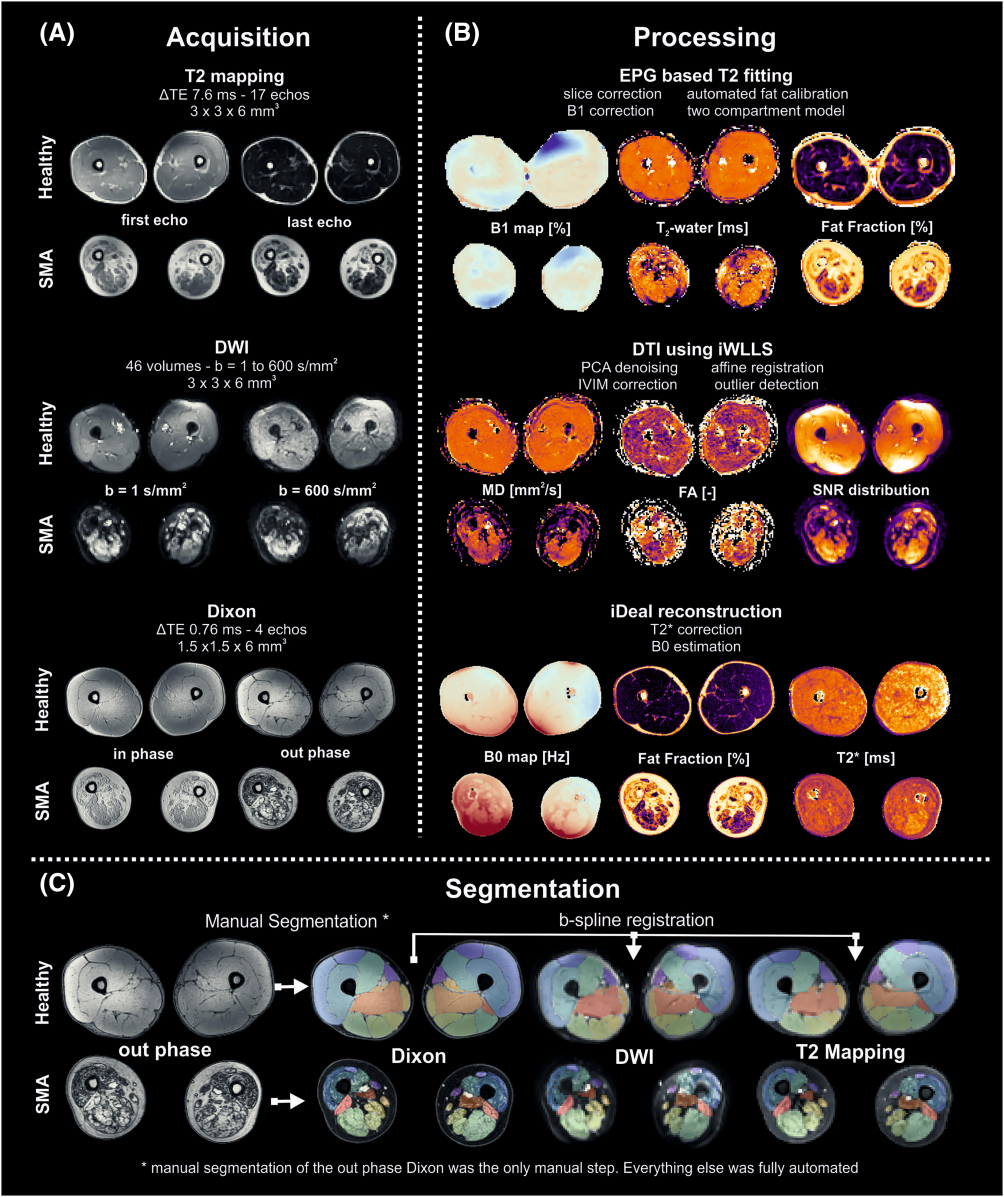
Data processing. Overview of the various steps involved in the acquisition and processing pipeline. (A) Example data for a healthy volunteer and a SMA patient for all three qMRI methods. (B) A summary of the processing steps and example parameters obtained for each of the qMRI methods. (C) Overview of the muscle segmentation. Muscle are manually segmented using the out‐phase Dixon image. Then segmentation is transferred to the DTI and T2 mapping data using b‐spline registration. DTI, diffusion tensor imaging; DWI, diffusion‐weighted imaging; FA, fraction anisotropy; MD, mean diffusivity; SMA, spinal muscular atrophy; TE, echo time

All MR data were processed using QMRITools for Mathematica (mfroeling.github.io/QMRITools).^29^ The processing steps for each method are summarized in Figure 2. Before processing all data were visually inspected for artefacts and data quality. The Dixon data were processed using an iterative decomposition of water and fat with echo asymmetry and least squares estimation (IDEAL)^30^ with B0 and T2* estimation. The T2‐mapping data were processed using an extended phase graph (EPG) fitting approach considering inhomogeneous B1^+^.^31^ This method accounts for different T2 relaxation times for the water and fat components with the T2 of the fat component fixed to a value calibrated on the subcutaneous fat. The diffusion data were processed using an fitting method (iWLLS) with REKINDLE outlier detection,^32^ taking intravoxel incoherent motion into account.^33^ Before tensor estimation the data were denoised using a principal component analysis method,^34^ after which data were corrected for subject motion and eddy current distortion using affine registration. A detailed overview of all processing steps and the multicenter reproducibility of the methods can be found in Schlaffke et al.^35^

Since the T2 and the DTI parameters have a bias with increasing fat contribution,^21^, ^25^ simulations were performed to estimate this effect.^21^, ^26^ The simulations were performed using multi‐compartment extensions of the used models with each compartment simulated as shown in Figure 1. For a detailed description of all simulations, refer to supplemental file (**S1**) (see the supporting information for this article). For estimation of the bias in T2 due to fat infiltration, fat fractions were simulated from 0% to 100% fat with a fixed known value for the T2 of fat of 180 ms and a T2 of water in fat (10%) of 20 ms.^36^, ^37^ T2 of fat was based on the calibration values of our data, T2 of water in fat on literature values and EPG T2 fitting of subcutaneous fat on spectroscopy experiments.^37^, ^38^, ^39^ With increasing fat fraction, the water component for fat creates a bias in the estimated T2 of muscle, since water T2 is modeled as a single T2 water value (see Figure 1). Similarly, in the estimation of tensor parameters, the water component of fat creates a bias in MD and FA. Here, this compartment is assumed to have isotropic diffusion (FA = 0) with an MD of 0.8 mm^2^/s. With an increasing fat fraction the water signal decreases causing a decrease in SNR as well as a stronger contribution of the water component of fat.^21^, ^25^

### 2.5 Muscle segmentation

The in‐phase or out‐phase images and fat images of the four‐point DIXON sequence were used for manual segmentation of individual muscles in the image stack. Open‐source software (ITK‐SNAP version 3.6)^40^ was used for segmentation on the 25 slices per image stack. Where the integrity of the muscle was still clear, muscles were annotated every third slice and then interpolated to fill in the missing slices. However, in more affected subjects with significant fat infiltration and atrophy, muscles had to be discriminated slice by slice. In any case of incongruency because of deviating anatomy in severely affected subjects, two authors (LO and MF) segmented the slices individually and reached a consensus. In total, 12 muscles per leg were partially apparent in the image stack. The demarcated regions of interest were then used to retrieve the quantitative information from the other related sequences. To transfer the DIXON‐based segmentations to the T2 and DTI data, a combined rigid and b‐spline image registration was used (Elastix).^41^ Muscles with a volume smaller than 10 voxels (67.5 mm^3^) were omitted from analyses.

### 2.6 Statistical analysis

Statistical analysis was performed using SPSS version 25 for Windows (SPSS Inc., Chicago, IL, USA). Differences between patients and controls were assessed by means of an independent Student's t‐test with Welch's correction. MR parameters were averaged across muscles per subject. Subjects were classified as either “case” for patients or “control”. The mean difference between cases and controls was converted into a standardized mean difference according to Hedge's g in order to compare effect sizes across qMRI parameters. For multiple group comparisons (ie, controls, SMA type 2 and SMA type 3), we used a one‐way ANOVA. Due to nonnormality of the data, associations between clinical and imaging parameters were evaluated using Kendall's tau correlation coefficient and associations were further explored with multiple linear regression. Results were considered significant when *P* < .05; we did not correct for multiplicity due to the exploratory nature of the analysis. Codes and data are available upon request to any qualified investigator.

## 3 RESULTS

### 3.1 Study population

We obtained datasets of 31 patients with SMA with a mean age of 29.6 (range 7.6‐73.9) years and of 20 controls with a mean age of 37.9 (17.7‐71.6) years. Minors could not be matched to controls. Fifteen patients had SMA type 2, seven type 3a and nine type 3b. Mean HMFSE score was 5 points for type 2 and 34 points for type 3. Patients’ characteristics, muscle strength and motor function mean values are reported in Table 2.

**TABLE 2:** Baseline demographics and characteristics. Clinical characteristics of study participants

	Type 2	Type 3a	Type 3b	Controls
n	15	7	9	20
Sex (M:F)	6:9	3:4	6:3	8:12
Age in years (range)	24.9 (7‐73)	23.5 (7‐49)	42.0 (18‐55)	37.9 (17‐71)
Mean disease duration in months (SD; range)	276 (172; 82‐661)	265 (202; 69‐581)	362 (180; 77‐661)	‐
Ambulatory status				
Ambulatory, n (%)	‐	3 (43%)	8 (89%)	20 (100%)
Nonambulatory, n (%)	15 (100%)	4 (57%)	1 (11%)	‐
Clinical measurements				
Mean MRC sum score (SD)	110 (34.4)	151 (43.7)	177 (38.8)	229 (2)
Mean MRC score lower extremities (SD)	11 (4.8)	15 (6.4)	17 (6.8)	30 (−)
Mean HFMSE score (SD; range)	5.9 (10; 0‐36)	28.4 (22.8; 0‐53)	40.0 (18.0; 2‐63)	‐

Abbreviations: F, female; HFMSE, Hammersmith Motor Function Scale Expanded; M, male; MRC, Medical Research Council; N, number; SD, standard deviation.

### 3.2 MR acquisition and processing

Two datasets of patients were excluded from final data analysis because the image quality was insufficient, resulting in 49 datasets. All muscle groups in controls and 58% of muscles in patients were successfully segmented (a total of 430 muscles: 34 rectus femoris, 29 vastus medialis, 23 vastus lateralis and 15 vastus intermedius muscle; 44 *m. semimembranosus*, 44 *m. semitendinosus*, *m. biceps femoris* long and short head 43 and 38, respectively; *m. adductor magnus* 38, the adductor longus muscle 44, and gracilis 41 and sartorius muscle 37).

### 3.3 Fat infiltration across age categories

**FIGURE 3:**
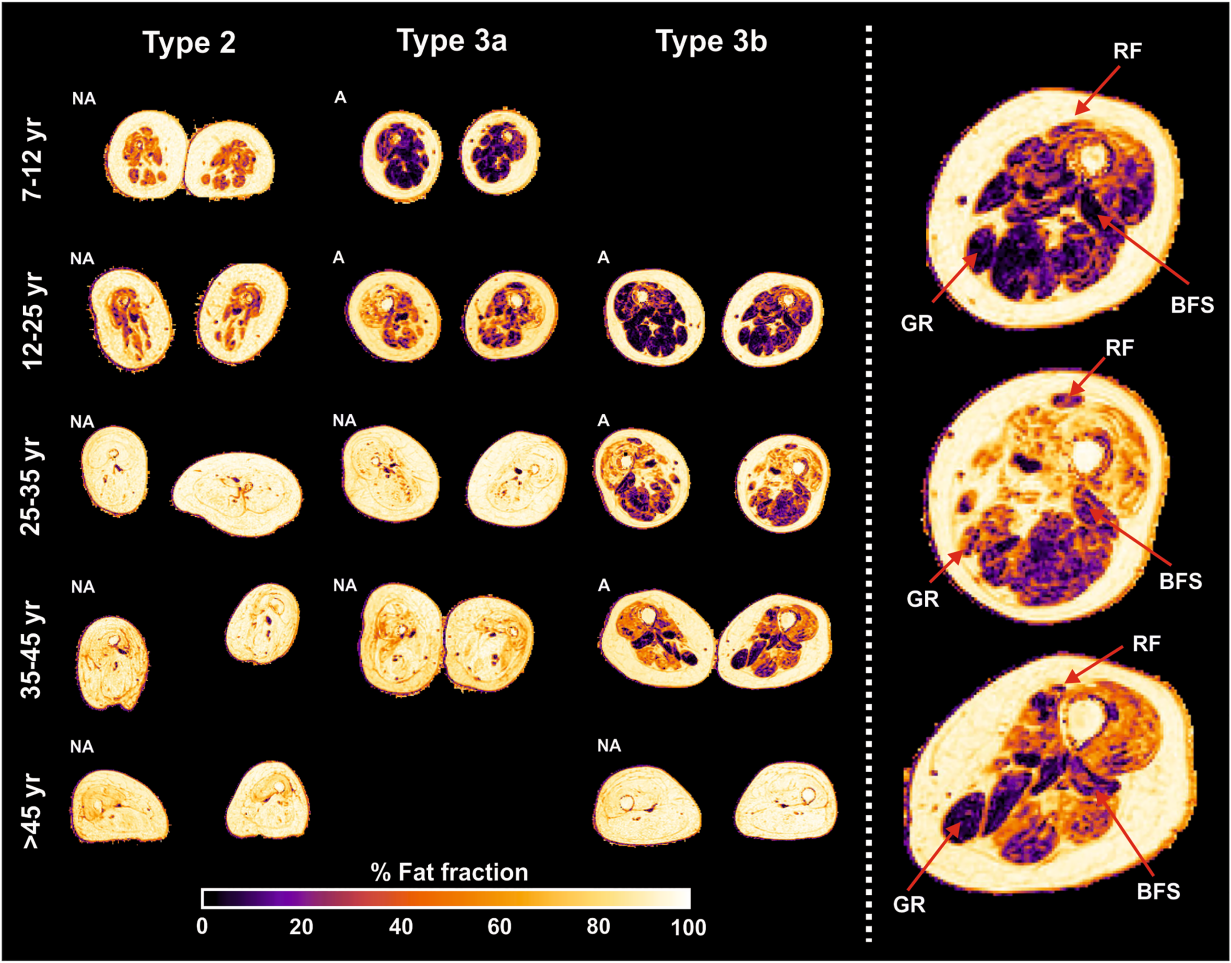
Overview of fat infiltration stratified for SMA type and age. Images of the thigh in the transversal plane of the middle section of the image stack. The images are categorized into age category for distinct SMA types. The heat bar beneath indicates a scale from 0% to 100%. The right column presents a magnification of the left leg of type 3b patients. The red arrows point at the annotated muscles, which seem relatively spared. A, ambulant; BFS, biceps femoris, short head; GR, gracilis; NA, nonambulant; RF, rectus femoris

Figure 3 summarizes patterns of fatty infiltration stratified for SMA type. Patients with SMA type 2 show generalized atrophy and fatty infiltration starting at a young age, underlined here by images of patients aged 7 years and older, and thickening of the subcutaneous fat layer. The pattern in types 3a and 3b appears more gradual, as fat infiltration of individual muscle groups was more pronounced from the age of 10 years and increased with age. Fat infiltration showed a characteristic pattern with predominant involvement of the anterior compartment, in particular the vasti muscles, whereas the *M. rectus* femoris remained identifiable when the vasti had already perished. The pattern of fat infiltration of the posterior compartment showed more heterogeneity. The short head of the biceps femoris, *m. semimembranosus*, *M. gracilis* and *m. sartorius* are the last muscles involved in the process of fatty replacement. The adductor magnus is affected at a relatively early stage while the *m. adductor longus* is relatively spared. From the start of puberty, muscle images of patients with SMA type 3a resemble type 2 more than type 3b. Complete fatty replacement with an intact fascia occurs in types 2 and 3a before the age of 30 years, but only after the age of 45 years in type 3b.

### 3.4 Comparison of groups

**FIGURE 4:**
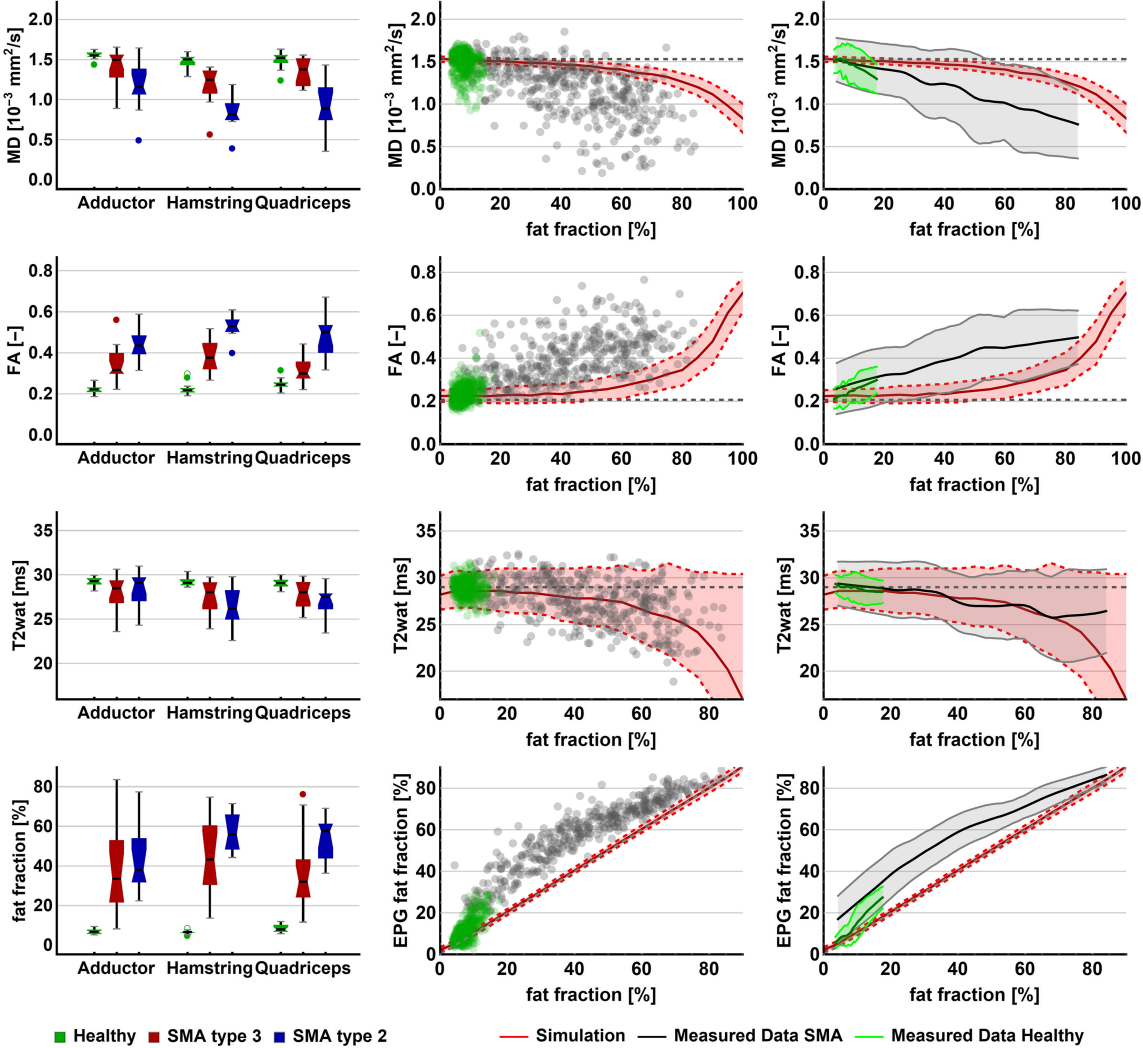
qMRI per muscle group and relations between qMRI parameters. The left panel shows boxplots for the qMRI parameters for the distinct muscle groups for healthy controls, SMA type 2 and SMA type 3. In the middle and right panels, each of the qMRI parameters is plotted against fat fraction. In the middle column, each data point represents a measurement of individual muscles of all subjects with SMA (gray) and control subjects (green). In the right column, the data points are reduced to an average using local regression and 95% CI (shaded area). The red line represents the association based on simulations of increasing fat fraction, theoretically ranging to 100. The gray and green lines depict the data of this study. CI, confidence interval; FA, fractional anisotropy; MD, mean diffusivity

7The mean values per qMRI parameter for patients and controls are given in Table 3. The individual muscles are now allocated to a specific muscle group; either quadriceps, hamstrings or adductors, with the exception of the m. sartorius and *M. gracilis*. Figure 4 (left column) depicts each qMRI measure for the distinct muscle groups for type 2 and type 3 patients versus controls. The comparison of type 2 and type 3 is based on measurements of seven patients with type 2 and 12 patients with type 3 in which one or more muscles of the quadriceps could be identified, and of the hamstrings in 23 patients (11 of whom were type 2 and 12 type 3) and adductor group in 25 patients (12 of whom were type 2 and 13 type 3).

**TABLE 3:** Descriptive statistics of the dataset. Cross‐sectional statistics of differences in qMRI outcomes between the SMA and control cohort. All reported values are in mean ± standard deviation

QMRI	SMA	CONTROLS	
FF‐DIXON (%)	47.6 ± 17.4	7.6 ± 1.5	mean difference: −40.0; 95% CI [−47.0 to −33.0] standardized mean difference: 3.06; *P*‐value <0.001
T2 (ms)	27.3 ± 1.5	28.9 ± 0.4	mean difference: 1.54; 95% CI [0.9 to 2.2] standardized mean difference: −1.38; *P*‐value <0.001
DTI ‐ FA	0.41 ± 0.09	0.24 ± 0.03	mean difference: −0.17; 95% CI [−0.2 to −0.1] standardized mean difference: 2.41; *P*‐value <0.001
DTI – MD (mm^2^/s)	1.13 ± 0.28	1.47 ± 0.10	mean difference: 0.34; 95% CI [0.2 to 0.5] standardized mean difference: −1.54; *P*‐value <0.001

Abbreviations: CI, confidence interval; DTI, diffusion tensor imaging; FA, fractional anisotropy; FF, fat fraction; MD, mean diffusivity; qMRI, quantitative magnetic resonance imaging; SMA, spinal muscular atrophy. Standardized mean difference according to Hedge's g.

### 3.5 Differences in fat fraction between groups

Mean fat fraction of all upper leg muscles was 7.6% ± 1.5% in controls and 47.6% ± 17.4% in patients (*P* < .001). Overall, fat fraction was higher in type 2 than in type 3 (51.5% ± 15.1% vs. 36.7% ± 20.5%, *P* < .001). Type 2 exhibits higher averages of fat fraction over all three muscle groups, yet only the hamstrings demonstrate a significantly lower fat fraction for type 3 (*P* = .012). The anterior compartment is most conspicuously fat‐infiltrated in all patients, with a mean fat fraction of 57.4% ± 10.1% in type 2 and 46.1% ± 20.3% in type 3 (*P* = .12). The adductor group has the lowest mean fat fraction in type 2 with 41.8% ± 14.9%. The posterior and middle compartments exhibit a comparable average fat fraction in type 3 (36.4% ± 20.0% and 36.9% ± 22.3%). On the level of individual muscles, the *m. adductor longus* has the lowest fat fraction in SMA. This contrast is most apparent in type 2 (29.1% ± 16.1%), where the fat fraction of all other muscles exceeds 50%. In type 3, the *m. semimembranosus* was the least affected (30.3% ± 14.5%).

### 3.6 Differences in T2 between groups

Mean T2, averaged over all muscles, is close in range for patients (27.3 ± 1.5) versus controls (28.9 ± 0.4) however significantly different (*P* < .001). For the distinct muscle groups, T2 does not show uniform differences for types 2 and 3 across muscle groups (hamstrings: *P* = .14; quadriceps: *P* = .26; adductors: *P* = .56).

### 3.7 Differences in DTI parameters between groups

MD was lowered in patients (1.13 ± 0.28) versus controls (1.47 ± 0.10, *P* < .001). When we compared patients, MD was significantly lower in type 2 versus type 3 for quadriceps and hamstrings (both *P* = .001), but not different for the adductors (*P* = .093). FA was higher (0.41 ± 0.09) in patients compared with controls (0.24 ± 0.03, *P* < .001). FA increased in type 3 and was highest in type 2 for all muscle groups (all *P* < .05).

### 3.8 The effect of fat fraction on T2 and DTI parameters

An increasing fat fraction introduces a bias in T2 and diffusion parameters, as we outlined in the Methods section. Consequently, we compared our measured data with simulations of this effect, as shown in Figure 4 (the two columns on the right). Theoretically, the effect of an increasing fat fraction would result in a gradual decrease of the T2 signal, a decrease in MD and an increase in FA. Our data show that T2 of fat‐infiltrated muscles followed the predicted T2 decrease. A decrease of MD was seen together with an increase of FA, both of greater magnitude than the predicted effect of partial volume effects of fat. Overall, we observed that the estimated EPG fat fraction was different from the estimated DIXON fat fraction.

### 3.9 Association of qMRI with clinical measurements

**FIGURE 5:**
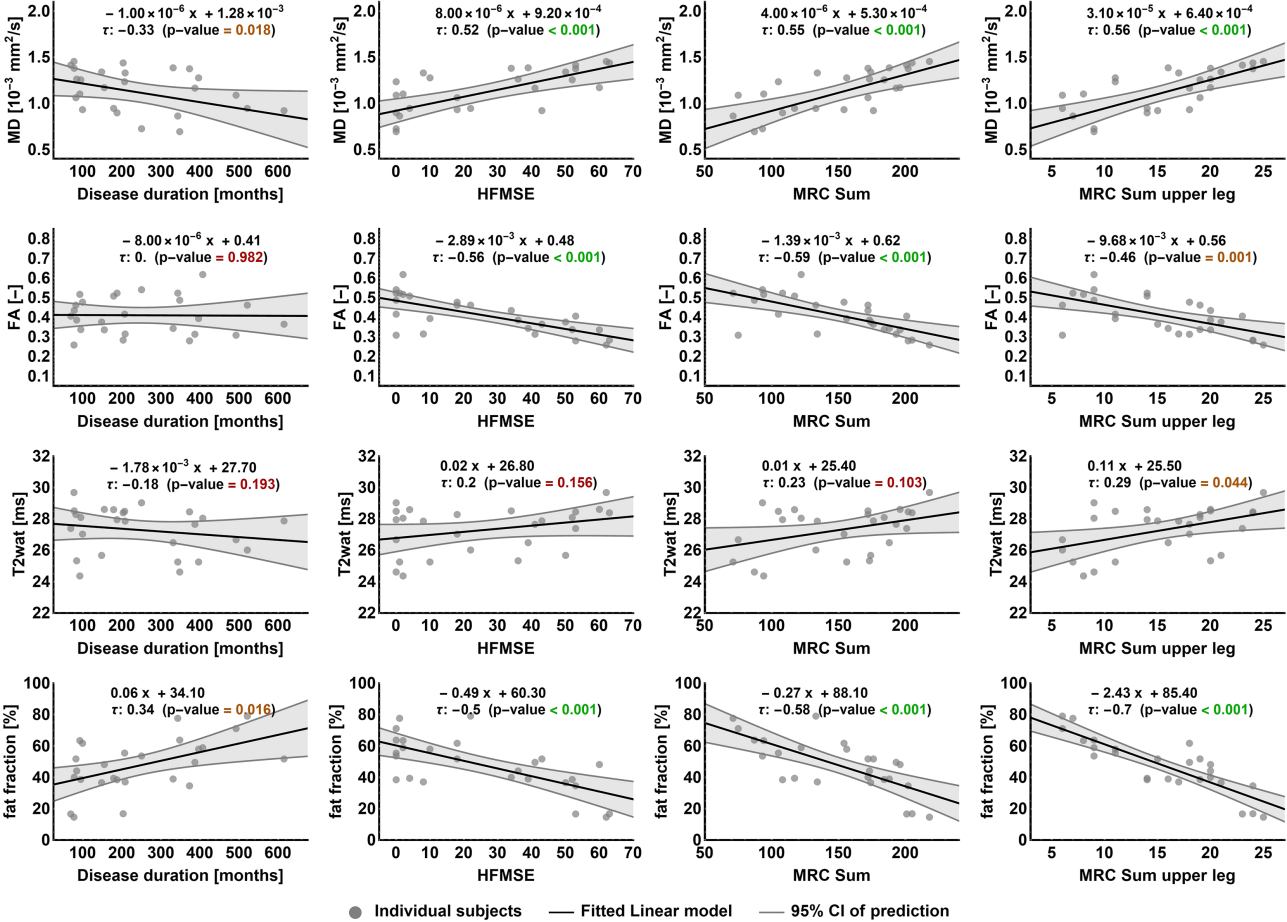
qMRI and relation with clinical measures. Each of the qMRI parameters (rows) is plotted against the following clinical outcome measures (columns); disease duration (in months), HFMSE score, MRC sum score and MRC sum score of the upper leg. Data points represent the average value of all muscles per patient per qMRI parameter plotted against patient's clinical score. The correlation formula, Kendall's tau correlation coefficient and the *P*‐value (significance level set at <0.05) are shown per correlation plot. HFMSE, Hammersmith Motor Function Scale Expanded, MRC, Medical Research Council

We explored each of the qMRI parameters in relation to clinical measures (Figure 5). Disease duration is not a direct reflection of disease severity but, given the progressive nature of SMA, is a determinant. We found a significant moderate correlation of disease duration with fat fraction (τ = 0.34, *P* = .016) and MD (τ = −0.33, *P* = .018), but not with FA (τ = 0.02, *P* = .982) or with T2 (τ = −0.18, *P* = .193).Motor function measured by the HFMSE scale showed a negative, moderate correlation with FA (τ = −0.56, *P* < .001) and fat fraction (τ = −0.5, *P* < .001). We observed a positive moderate correlation of MD (τ = 0.52, *P* < .001) and HFMSE but not of T2 (τ = 0.2, *P* = .156) with HFMSE. There was a moderate significant correlation of muscle strength represented by the MRC sum score with FA (τ = −0.59, *P* < .001), with fat fraction (τ = 0.58, *P* < .001) and MD (τ = 0.55, *P* < .001). We did not find a significant correlation with T2 (τ = 0.23, *P* = .103). When we plotted the MRC sum score of the hamstrings, adductor and quadriceps muscles against the qMRI parameters, we observed similar trends (see Figure 5, right column), with the strongest relationship with fat fraction (τ = 0.7, *P* < .001). This correlation was similar to the correlation of contractile muscle cross‐sectional area (CSA). For a detailed overview of CSA and volume of thigh muscle, and correlation with strength, we refer to supplementary file S2.

FA changes are partly driven by fat infiltration, but nonetheless FA has a significant contribution to the prediction of thigh muscle strength independent of fat fraction. Multiple linear regression analysis showed a significant contribution of fat fraction to the MRC sum score of upper leg with an R^2^ of 0.76, whereas the R^2^ of only MD or FA is 0.50 or 0.34, respectively. Adding both FA and fat fraction to the model increased the R^2^ to 0.80, while the addition of MD did not contribute significantly.

## 4 DISCUSSION

This study on a relatively large number of patients of different ages and disease severities outlines leg muscle involvement across the clinical spectrum of SMA and adds to accumulating experience with qMRI measures in neuromuscular diseases. DTI further elucidates distinct properties of skeletal muscle in SMA. The correlation of qMRI and clinical parameters suggest potential as a biomarker for disease progression or treatment effect. Here, we extend the previous descriptions of muscle involvement in SMA with a diverse cohort, rising from 7 to 73 years of age. The patterns of muscle involvement and relative muscle sparing were comparable with previous reports such as the *m. adductor longus*, *M. gracilis*, *m. sartorius*, and the short head of the *m. biceps femoris* and *m. semimembranosus.* ^12^, ^13^, ^18^, ^19^, ^42^ Strikingly, the severe involvement of the quadriceps did not impede some patients with type 3 to walk. Selective vulnerability of muscle is a hallmark of SMA but whether this is explained by anatomical or biochemical (ie, related to SMN deficiency)^43^, ^44^ differences between muscle groups remains to established. The progressive nature of SMA at muscle level is reflected by the more severe fatty infiltration of muscles in older patients. Similarly, patients with type 2 had earlier onset of fatty infiltration and therefore a higher fat fraction in all muscle groups than patients with type 3 at a similar age. The inclusion of older and severely affected patients allowed a cross‐sectional description of qMRI across the full spectrum of SMA, which has not been described before. However, end‐stage fatty infiltration resulted in the exclusion of these muscles from quantitative analysis because muscle was either no longer present, impossible to segment, unidentifiable, or under a volume of 10 voxels. Nonetheless, our data included muscles up to 80% of fat infiltration. Although little muscle was present, we could still establish a correlation between properties of remaining muscle and functional data. The use for longitudinal purposes, ie, to study the natural history of the quality of muscle tissue or the effects of treatment, therefore seems limited to young children with SMA type 2 and adolescents and young adults with SMA type 3.

Elevated T2 signal has been reported in neuromuscular disorders that trigger inflammatory processes in muscles, such as in DMD. Since the leukocyte infiltrates observed in DMD are not a hallmark of SMA, we did not expect similar changes in SMA. Nevertheless, a previous study reported an increased T2 in the upper leg in SMA patients, ranging from 34.3 to 41.3 ms, but this cannot be directly compared with our data as the authors used a multi‐exponential signal model that does not account for EPG effects.^23^ Moreover, Bonati et al found a strongly increased T2 in SMA patients in comparison with controls, exceeding 60 ms, whereas our data ranged to 31 ms at most.^24^ We think that these differences are explained by methodological issues. In contrast to previous studies, we applied a two‐compartment fit for T2 mapping, which allowed a better estimation of water T2 in the muscle, taking into account the partial volume effects of fat and the fat‐independent behavior of muscle water T2.^45^ After correction for the increase of T2 caused by fatty infiltration,^46^ we found a decrease in T2 that almost exactly follows the simulation of T2 against an increasing fat fraction, similar to a previous study.^26^ Therefore, we conclude that a decrease of T2 as we perceived is solely related to partial volume effects caused by the amount of fat infiltration and that the T2 signal increase in itself does not indicate any pathological process. However, if the T2 relaxation of water in fat had a higher value of ~ 30 ms instead of the assumed 20 ms, part of the decreased T2 could potentially be attributed to pathological processes.

DTI analysis showed lowered MD and increased FA values in patients with SMA. As fat fraction is also a confounder on DTI, we repeated the simulation experiment in which we plotted MD and FA against an increasing fat fraction.^21^, ^25^ After correction, our data illustrates stronger effects, ie, a further decrease of MD and increase of FA than could be explained based on partial volume effects alone. If the model wrongfully contributed the water component of fat to the muscle compartment, the results would be an underestimation of the situation and this would have led to even stronger effects. This suggests that we are indeed measuring disease‐specific processes and that MD/FA measures behave independently of an increasing fat fraction. The latter also becomes apparent when we look at the relation of qMRI measures and clinical scores. Fat fraction strongly and FA and MD moderately correlate with thigh muscle strength. However, FA and fat fraction together improve the prediction of muscle strength and, despite its modest contribution, suggest that FA adds to the prediction of MRC sum score independently of fat infiltration. This shows that although FA is strongly correlated with fat infiltration the diffusion parameters do provide additional information on muscle status.

An association of strength or function with DTI parameters has not been consistently found in DMD or LGMD.^47^, ^48^ This may be explained by differences in muscular pathology but could also be due to methodological differences, including improved processing or segmentation of individual muscles rather than of muscle groups that could also potentially have benefited determining the relation between clinical parameters and image.The decrease of MD could indicate that the cellular compartment is subject to shrinkage, which is supported by the elevation of FA. This may be explained by atrophy of muscle fibers. The alternative explanation, in which the decrease of MD reflects changes in viscosity of the extracellular space due to fibrosis^26^ is less likely, since it would be accompanied by an invariable FA, as the cellular dimension would not change. Therefore, we hypothesize that our DTI findings indicate ongoing muscular atrophy, fitting the histopathological characteristics of muscle tissue rather than fibrosis. In muscle biopsies from patients with SMA, round atrophic fibers are encountered in combination with large hypertrophic fibers,^49^ which is compatible with an increased FA. Our data show that the changes in MD and FA precede fat infiltration. As fat infiltration is considered as an end‐stage effect, the chances in restoring muscle function lie in the phase prior to fatty degeneration. A tool that is able to capture this transition zone, as we hereby demonstrate with MD/FA measures, may be very useful to monitor treatment effects in individual patients. Determining whether FA and MD are markers of alterations in muscle that precede fat infiltration should be a topic of future research. Follow‐up data of patients will provide more insight into its potential in capturing disease. The use of qMRI measures in patients undergoing treatment would provide additional information on its sensitivity when monitoring early therapy effects.

Our study has several limitations. First, the variation in ages and disease severity is in itself an impeding factor for general statements. Furthermore, longitudinal data need to confirm or counterfeit the cross‐sectional results from this study. A bigger sample size would obviously benefit statistical power, yet the number of patients is acceptable for a rare disease. To allow the inclusion of young children we kept the scan time minimal, which compromises resolution and SNR, yet no data had to be excluded due to motion artifacts. Furthermore, the high end of fat infiltration is pushing the limits of fat‐suppression techniques and its reliability. However, each of the methods includes visual and quantitative quality checks of the data, ie, the b‐0 map for DIXON, b‐1 for T2 mapping, and SNR for DTI. Unfortunately, we were not able to find corresponding control subjects for matching to the very young patients. Although we developed a fully automated pipeline for processing the MRI data uniformly and reproducibly, manual segmentation was still needed, which is both prone to error and time‐consuming. Lastly, there was a notable difference between the fat fraction estimated by DIXON and by EPG which needs further investigation as a clear explanation for this is currently lacking.

In conclusion, we have provided qMRI measurements of skeletal muscle in a large cohort of SMA patients widely ranging in age and disease severity. This study depicts the natural disease course of muscle tissue. The MRI protocol we used has demonstrated high temporal stability and multicenter reproducibility and proved tolerable even by very young children. This study shows that DTI parameters complement existing MR protocols that have focused on T2 signal and fat fraction, and manifest good correlation with clinical measures. We disclose abnormal DTI properties of skeletal muscle in SMA that have not been described before. DTI serves as a distinct parameter that can be monitored alongside and independent to the process of fat infiltration. This demonstrates the potential of quantitative MRI markers to study SMA pathogenesis and to monitor disease progression and treatment effects in muscle.

## COMPETING INTEREST

WLP served as an ad hoc member of the scientific advisory board of Biogen, Avexis and as a member of the Branaplam data monitoring committee (Novartis). BB performed consultancy activities for Scholar Rock and Cytokinetics. WLP and BB report grants from Prinses Beatrix Spierfonds, grants from Stichting Spieren voor Spieren, grants from Vriendenloterij. The other co‐authors report no competing interest.

## Supporting information


**Data S1.** Supporting information

**Data S2.** Supporting information
