# Supplementary material for: Quantitative MRI of skeletal muscle in a cross‐sectional cohort of patients with spinal muscular atrophy types 2 and 3
Source: NMR Biomed. 2020 Jul 18;33(10):e4357. doi: 10.1002/nbm.4357 (PMC7507182; doi:10.1002/nbm.4357)
Supplement: Supplementary file 1 — Data S1. Supporting information [file NBM-33-e4357-s001.docx]

**Supplementary files – S1 Simulations DTI and T2 (EPG-T2) explained**

# DTI simulations

Simulations were performed similar to

1. Froeling M, Nederveen AJAJ, Nicolay K, Strijkers GJGJGJ: DTI of human skeletal muscle: The effects of diffusion encoding parameters, signal-to-noise ratio and T2 on tensor indices and fiber tracts. NMR Biomed 2013; 26:1339–1352.
2. Damon BM: Effects of image noise in muscle diffusion tensor (DT)-MRI assessed using numerical simulations. Magn Reson Med 2008; 60:934–944.
3. **Acquisition parameters:**

Same as real data (see Table 1 of manuscript)

1. **Tissue parameters**

The tissue parameters that were used in the simulations were:

|  | **Muscle compartment** | **Fat compartment** |
| --- | --- | --- |
| Proton density | 0.9 | 0.1 |
| T1 relaxation [ms] | 1200 | 300 |
| T2 relaxation [ms] | 30 | 20 |
| Tensor eigen values [10-3 mm^2^/s] | 1.88; 1.46; 1.25 | 0.8; 0.8;0.8 |
| Derived MD and FA | 1.53; 0.21 | 0.8; 0.0 |
| Tensor eigen vector direction | Along z direction | Random |

1. **Simulation parameters**

- SNR value: 30
- Fat range: 0 to 100% with steps of 5%

1. **Simulation**

Per compartment (either muscle or fat) the signal was generated as:

$$S_{0}=\rho\left( 1-e^{\frac{-TR}{T1}} \right)e^{\frac{-TE}{T2}}$$

$$S_{ib}=S_{0}e^{-b{\vec{\mathbf{g}}}_{i}^{T}\mathbf{D}{\vec{\mathbf{g}}}_{i}}$$

Here TR is the repetition time, TE the echo time, T1 the longitudinal relaxation time, T2 the transverse relaxation time, and $\rho$ the proton density. The diffusion-weighted signal intensity $S_{ib}$ is related to the non-weighted signal intensity $S_{0}$ via the diffusion tensor $\mathbf{D}$, the b-value $b$ and the gradient directions ${\vec{\mathbf{g}}}_{i}$ (Eq. 2). The diffusion tensor $\mathbf{D}$ was decomposed into its eigenvalues $\lambda_{i}$ and eigenvectors ${\vec{\boldsymbol{\varepsilon}}}_{i}$ using

$$\mathbf{D}=\mathbf{E}\cdot\boldsymbol{\Lambda}\cdot\mathbf{E}^{T}$$

$$\mathbf{E}=\left[ \begin{matrix} \vdots& \vdots& \vdots\\ {\vec{\boldsymbol{\varepsilon}}}_{1} & {\vec{\boldsymbol{\varepsilon}}}_{2} & {\vec{\boldsymbol{\varepsilon}}}_{3} \\ \vdots& \vdots& \vdots\end{matrix} \right]$$

$$\boldsymbol{\Lambda}=\left[ \begin{matrix} \lambda_{1} & 0 & 0 \\ 0 & \lambda_{2} & 0 \\ 0 & 0 & \lambda_{3} \end{matrix} \right]$$

The combined signal from water and fat was defined as

$$S_{ib,total}={\left( 1-f \right)*S}_{ib,muscle}{+ f*S}_{ib,fat}$$

With the fat fraction $f$ ranging from 0 to 1.

In total 10000 signal were generated for each fat fraction after which Rician noise was added. Data was fitted identical to the acquired MRI data as described in the manuscript.

# EPG T2 simulations

The EPG signals were simulated according to

1. Marty B, Baudin PY, Reyngoudt H, et al.: Simultaneous muscle water T2and fat fraction mapping using transverse relaxometry with stimulated echo compensation. NMR Biomed 2016; 29:431–443.
2. **Acquisition parameters:**

Same as real data (see Table 1 of manuscript)

1. **Tissue parameters**

The tissue parameters that were used in the simulations were:

|  | **Muscle compartment** | **Fat compartment  water** | **Fat compartment  fat** |
| --- | --- | --- | --- |
| Relative size | - | 10% | 90% |
| T1 relaxation [ms] | 1200 | 1000 | 500 |
| T2 relaxation [ms] | 30 | 20 | 175 |

1. **Simulation parameters**

- SNR value: 50
- B1 value: 100%
- Fat range: 0 to 100% with steps of 5%

1. **Simulation**

The measured signal $\boldsymbol{S}$ with size $N_{echo}$can be approximated using a bi-component EPG model where the signal can be defined as

$$\hat{\boldsymbol{S}}=w \boldsymbol{S}_{water}+f \boldsymbol{S}_{fat}=\left[ \begin{matrix} \boldsymbol{S}_{water} & \boldsymbol{S}_{fat} \end{matrix} \right]\left[ \begin{matrix} w \\ f \end{matrix} \right]$$

With $w$ and $f$ the water and fat signal amplitudes which in the simulation were defined as $w=1-f$. The signal at each echo of the water and fat component are $\boldsymbol{S}_{water}$ and $\boldsymbol{S}_{fat}$ ,respectively, and are defined as

$$\boldsymbol{S}_{water}= \sum_{m=1}^{M} EPG\left( T_{1,wat},T_{2,wat},B_{1},\Delta TE, N_{echo},\boldsymbol{\alpha}_{ex}^{m},\boldsymbol{\alpha}_{ref}^{m} \right)$$

$$\boldsymbol{S}_{fat}= \sum_{m=1}^{M} \left( 1-g \right)EPG\left( T_{1,fat},T_{2,fat},\boldsymbol{B}_{1}^{i},\Delta TE, N_{echo},\boldsymbol{\alpha}_{ex}^{m},\boldsymbol{\alpha}_{ref}^{m} \right)+g EPG\left( T_{1,watfat},T_{2,watfat},\boldsymbol{B}_{1}^{i},\Delta TE, N_{echo},\boldsymbol{\alpha}_{ex}^{m},\boldsymbol{\alpha}_{ref}^{m} \right)$$

With $\boldsymbol{\alpha}_{ex}$ and $\boldsymbol{\alpha}_{ref}$ the flip angle profiles along the slice direction of the excitation and refocusing pulses, respectively, and $m$ the number of samples along the slice profile and $g$ is the water fraction in fat which was 10%.

In total 10000 signal were generated for each fat fraction after which Rician noise was added. Data was fitted identical to the real data as described in the manuscript.
