# Supplementary material for: Quantitative MRI of skeletal muscle in a cross‐sectional cohort of patients with spinal muscular atrophy types 2 and 3
Source: NMR Biomed. 2020 Jul 18;33(10):e4357. doi: 10.1002/nbm.4357 (PMC7507182; doi:10.1002/nbm.4357)
Supplement: Supplementary file 2 — Data S2. Supporting information [file NBM-33-e4357-s002.docx]

S2 – **Cross-sectional area (CSA) and volumes of thigh muscle of SMA patients**

| **Patients** | **CSA**  **[cm^2^]** | **Contractile-CSA (ccsa) [cm^2^]** | **Volume (vol)**  **[cm^3^]** | **Contractile volume (cvol) [cm^3^]** |
| --- | --- | --- | --- | --- |
| P1 | 0 | 0 | 0 | 0 |
| P2 | 350,89 | 285,57 | 1774,46 | 1433,57 |
| P3 | 77,13 | 46,62 | 370,49 | 209,02 |
| P4 | 0 | 0 | 0 | 0 |
| P5 | 55,89 | 11,63 | 165,77 | 36,962 |
| P6 | 158,72 | 103,87 | 788,83 | 484,03 |
| P7 | 99,68 | 59,38 | 470,44 | 290,388 |
| P8 | 0 | 0 | 0 | 0 |
| P9 | 41,58 | 19,49 | 136,22 | 62,87 |
| P10 | 4,75 | 1,99 | 11,28 | 5,13 |
| P11 | 31,43 | 15,14 | 93,74 | 41,89 |
| P12 | 126,09 | 83,09 | 519,51 | 322,69 |
| P13 | 11,79 | 7,99 | 28,63 | 18,43 |
| P14 | 3,98 | 1,21 | 16,94 | 4,02 |
| P15 | 327,40 | 213,02 | 1512,70 | 957,38 |
| P16 | 171,65 | 83,95 | 810,16 | 367,15 |
| P17 | 20,88 | 14,51 | 70,91 | 45,80 |
| P18 | 69,86 | 40,12 | 318,52 | 170,54 |
| P19 | 190,28 | 161,48 | 839,85 | 693,63 |
| P20 | 6,39 | 1,88 | 20,03 | 5,98 |
| P21 | 13,19 | 6,05 | 28,60 | 12,98 |
| P22 | 59,69 | 39,38 | 233,78 | 145,88 |
| P23 | 84,02 | 43,23 | 349,23 | 164,47 |
| P24 | 65,14 | 52,54 | 281,55 | 220,01 |
| P25 | 208,82 | 108,02 | 932,28 | 474,73 |
| P26 | 15,64 | 6,66 | 41,05 | 17,37 |
| P27 | 65,75 | 43,00 | 195,99 | 121,06 |
| P28 | 36,65 | 14,80 | 137,20 | 53,83 |
| P29 | 69,59 | 27,54 | 311,01 | 104,62 |

**Correlation plots of (A) cross-sectional area (CSA); (B) contractile CSA (ccsa); (C) volume and (D) contractile volume (cvol) versus MRC sum score of upper legs.** The correlation formula, Kendall’s tau correlation coefficient and the p-value (significance level set at <0.05) are shown per correlation plot.

| **A** 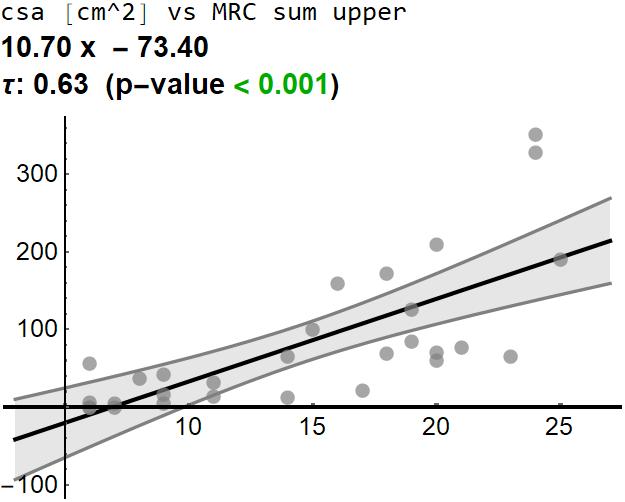 | **B** 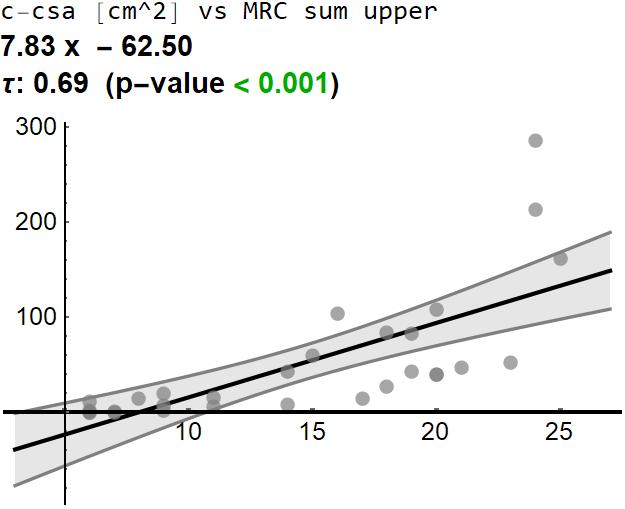 |
| --- | --- |
| **C** 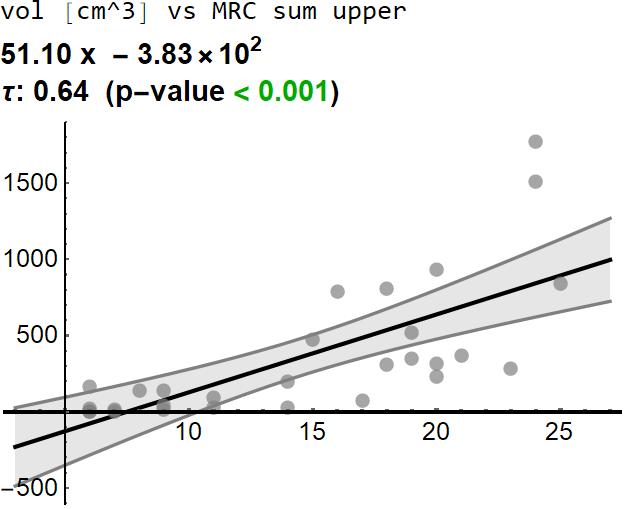 | **D** 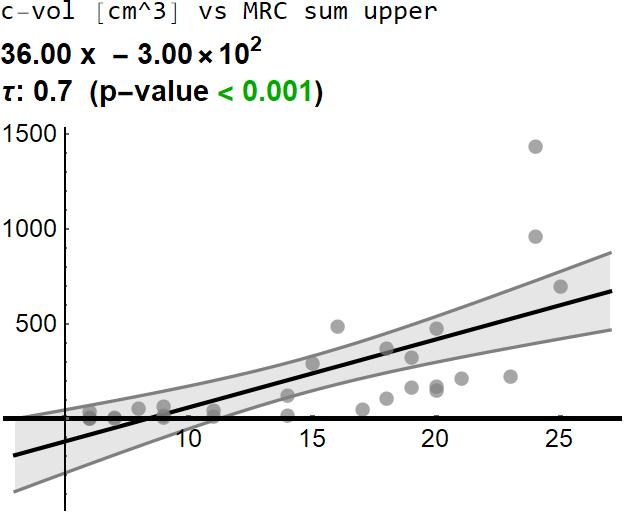 |
